# (1*S*,2*E*,6*R*,7a*R*)-2-Benzyl­idene-1,6-dihy­droxy-2,3,5,6,7,7a-hexa­hydro-1*H*-pyrrolizin-3-one

**DOI:** 10.1107/S1600536812018223

**Published:** 2012-04-28

**Authors:** F. L. Oliveira, K. R. L. Freire, R. Aparicio, F. Coelho

**Affiliations:** a Laboratory of Structural Biology and Crystallography, Institute of Chemistry, University of Campinas, CP6154, CEP13083-970, Campinas, SP, Brazil; b Laboratory of Synthesis of Natural Products and Drugs, Institute of Chemistry, University of Campinas, CP6154, CEP13083-970, Campinas, SP, Brazil

## Abstract

In the title compound, C_14_H_15_NO_3_, the conformation of the double bond was determined to be *E*, confirming the result obtained from two-dimensional NMR data. The five-membered rings of the pyrrolizine unit exhibit C-envelope conformations, with C atoms displaced from the mean planes formed by the remaining rings atoms by 0.1468 (15) and 0.5405 (17) Å. The mean planes of these rings (through all ring atoms) have a dihedral angle of 49.03 (10)°. In the crystal, mol­ecules are linked by O—H⋯O hydrogen bonds. The absolute configuration of the mol­ecule was established, as judged by the, as judged by the obtained values for the Hooft and Flack parameters.

## Related literature

For the preparation of the title compound, see: Freire *et al.* (2011 ▶). For the use of this type of compound as LFA-1 (Lymphocyte Function-Associated Anti­gen-1) inhibitors, see: Baumann (2007 ▶). For related structures, see: Oliveira *et al.* (2012*a* ▶,*b* ▶).
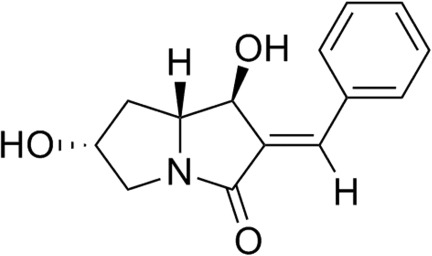


## Experimental

### 

#### Crystal data

C_14_H_15_NO_3_
*M* *_r_* = 245.27
Orthorhombic, 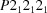

*a* = 6.5007 (3) Å
*b* = 13.6783 (7) Å
*c* = 13.8382 (7) Å
*V* = 1230.47 (11) Å^3^
*Z* = 4
Cu *K*α radiation
μ = 0.77 mm^−1^
*T* = 100 K
0.31 × 0.13 × 0.13 mm

#### Data collection

Bruker Kappa APEXII DUO diffractometer
Absorption correction: numerical (*SADABS*; Bruker, 2010 ▶) *T*_min_ = 0.924, *T*_max_ = 1.000
32528 measured reflections
2219 independent reflections
2203 reflections with *I* > 2σ(*I*)
*R*_int_ = 0.034

#### Refinement

*R*[*F* ^2^ > 2σ(*F* ^2^)] = 0.037
*wR*(*F* ^2^) = 0.098
*S* = 1.06
2219 reflections
165 parameters
H-atom parameters constrained
Δρ_max_ = 0.27 e Å^−3^
Δρ_min_ = −0.23 e Å^−3^
Absolute structure: Flack (1983 ▶) and Hooft *et al.* (2008 ▶); Hooft parameter = 0.01(2), 905 Bijvoet pairs
Flack parameter: 0.1 (3)

### 

Data collection: *APEX2* (Bruker, 2010) ▶; cell refinement: *SAINT* (Bruker, 2010 ▶); data reduction: *SAINT*; program(s) used to solve structure: *SHELXS97* (Sheldrick, 2008 ▶); program(s) used to refine structure: *SHELXL97* (Sheldrick, 2008 ▶); molecular graphics: *PLATON* (Spek, 2009 ▶); software used to prepare material for publication: *publCIF* (Westrip, 2010 ▶).

## Supplementary Material

Crystal structure: contains datablock(s) I, global. DOI: 10.1107/S1600536812018223/pv2526sup1.cif

Supplementary material file. DOI: 10.1107/S1600536812018223/pv2526Isup2.cml

Structure factors: contains datablock(s) I. DOI: 10.1107/S1600536812018223/pv2526Isup3.hkl

Additional supplementary materials:  crystallographic information; 3D view; checkCIF report

## Figures and Tables

**Table 1:** Hydrogen-bond geometry (Å, °)

*D*—H⋯*A*	*D*—H	H⋯*A*	*D*⋯*A*	*D*—H⋯*A*
O1—H1⋯O3^i^	0.84	2.04	2.776 (2)	147
O3—H3⋯O2^ii^	0.84	1.85	2.6810 (17)	168

Symmetry codes: (i) 
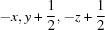
; (ii) 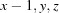.

**Fig. 1.**
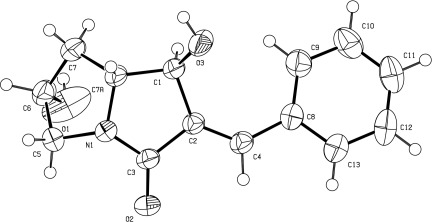
The molecular structure of the title compound with the atom numbering scheme. Displacement ellipsoids are drawn at the 50% probability level. H atoms are presented as small spheres of arbitrary radius.

**Fig. 2.**
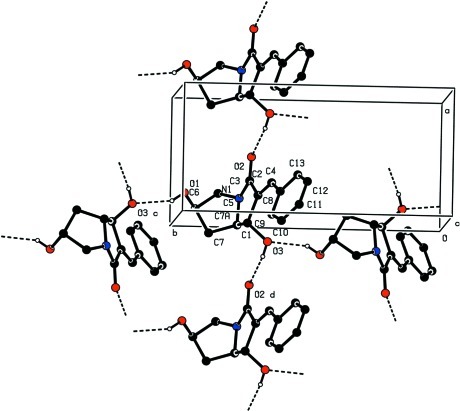
A view of the hydrogen bonding interactions (dotted lines) in the crystal structure of the title compound. H atoms non-participating in hydrogen-bonding were omitted for clarity.

## supplementary crystallographic information

### Comment

The title compound (Fig. 1) is a new asymmetric benzyl-pyrrolizidinone which has been synthesized from a chiral Morita-Baylis-Hillman adduct. It belongs to a class of compounds with potential pharmacological properties as mediators of the LFA-1 (lymphocyte function-associated antigen 1) function, particularly as anti-inflammatory agents and for the treatment of autoimmune diseases (Baumann, 2007). It has three defined stereocenters and a double bond with *E* configuration. The five membered rings N1/C3/C2/C1/C7A and N1/C5/C6/C7/C7A of the pyrrolizine moiety exhibit C2- and C5-envelope conformations, respectively, with C2 and C5 atoms displaced from the mean-planes formed by the remaining rings atoms by 0.1468 (15) and 0.5405 (17) Å, respectively. The mean planes of these rings have a dihedral angle of 49.03 (10)°. The configuration of the double bond determined by X-ray crystallography confirms the two-dimensional-NOESY NMR analysis. The crystal structure is stabilized by intermolecular hydrogen bonds (Tab. 1 and Fig. 2).

### Experimental

The title compound was prepared using a synthetic sequence previously described (Freire *et al.*, 2011) and purified by flash silica gel column chromatography (CH_2_Cl_2_:MeOH – solvent gradient: 100:0 to 95:05) to afford 0.14 g (as a white solid) in 76% yield, followed by recrystallization using the liquid-vapor saturation method. Subsequently, it was dissolved in ethanol and crystallized under the vapor pressure of ethyl ether (a less polar liquid), in a closed camera, thus allowing for the slow formation of good diffracting crystals.

### Refinement

The calculated Flack parameter was F = 0.1 (3) (Flack, 1983). Further analysis OF the absolute structure was performed with *PLATON* (Spek, 2009), using likelihood methods (Hooft *et al.*, 2008). The calculated value for the Hooft parameter was *y* = 0.01 (2), with a corresponding probability of 1x10^-100^ for an inverted structure. These results unequivocally indicate that the absolute structure has been correctly assigned. All H atoms were placed in calculated positions with O—H = 0.84 Å and C—H = 0.95, 0.99 and 1.00 Å for aryl, methylene and methyne H-atoms, respectively, and refined in the riding model approximation with *U*_iso_(H) = 1.5 *U*_eq_(O) or 1.2 *U*_eq_(C).

### Crystal data

**Table:**

C_14_H_15_NO_3_	*D*_x_ = 1.324 Mg m^−^^3^
*M**_r_* = 245.27	Cu *K*α radiation, λ = 1.54178 Å
Orthorhombic, *P*2_1_2_1_2_1_	Cell parameters from 2219 reflections
*a* = 6.5007 (3) Å	θ = 4.6–68.1°
*b* = 13.6783 (7) Å	µ = 0.77 mm^−^^1^
*c* = 13.8382 (7) Å	*T* = 100 K
*V* = 1230.47 (11) Å^3^	Rectangular, colourless
*Z* = 4	0.31 × 0.13 × 0.13 mm
*F*(000) = 520	

### Data collection

**Table:**

Bruker Kappa APEXII DUO diffractometer	2219 independent reflections
Radiation source: fine-focus sealed tube	2203 reflections with *I* > 2σ(*I*)
Graphite monochromator	*R*_int_ = 0.034
Bruker APEX CCD area–detector scans	θ_max_ = 68.1°, θ_min_ = 4.6°
Absorption correction: numerical (*SADABS*; Bruker, 2010)	*h* = −7→5
*T*_min_ = 0.924, *T*_max_ = 1.000	*k* = −16→16
32528 measured reflections	*l* = −16→16

### Refinement

**Table:**

Refinement on *F*^2^	Secondary atom site location: difference Fourier map
Least-squares matrix: full	Hydrogen site location: inferred from neighbouring sites
*R*[*F*^2^ > 2σ(*F*^2^)] = 0.037	H-atom parameters constrained
*wR*(*F*^2^) = 0.098	*w* = 1/[σ^2^(*F*_o_^2^) + (0.0574*P*)^2^ + 0.2433*P*] where *P* = (*F*_o_^2^ + 2*F*_c_^2^)/3
*S* = 1.06	(Δ/σ)_max_ < 0.001
2219 reflections	Δρ_max_ = 0.27 e Å^−^^3^
165 parameters	Δρ_min_ = −0.23 e Å^−^^3^
0 restraints	Absolute structure: Flack (1983) and Hooft *et al.* (2008); Hooft parameter = 0.01(2), 905 Bijvoet pairs
Primary atom site location: structure-invariant direct methods	Flack parameter: 0.1 (3)

### Special details

**Table:**

Experimental. [α]D^20^ + 40 (c 1, MeOH); IR (Film, ν_max_): 3427, 3195, 2940, 2855, 1668, 1634, 1493, 1424, 1268, 1156, 1067 cm^-1^; ^1^H NMR (400 MHz, CD3CN) δ 1.30 (m, *J* = 13.8, 9.1, 5.4 Hz, 1H, H-14 A), 2.38 (m, *J* = 13.8, 6.8 Hz, 1H, H-14B), 3.27 (dd, *J* = 12.3, 6.1 Hz, 1H, H-2 A), 3.56 (dd, *J* = 12.3, 3.3 Hz, 1H, H-2B), 3.69 (ddd, *J* = 9.1, 7.4, 1.8 Hz, 1H, H-10), 4.47 (qd, *J* = 6.1, 3.3 Hz, 1H, H-1 A), 4.91 (dd, *J* = 1.8 Hz, 1H, H-11), 7.35 (d, *J* = 2.1 Hz, 1H, H-5), 7.41 (m, 3H, Ph), 7.79 (m, 2H, Ph); ^13^C NMR (62.5 MHz, (CD_3_)_2_CO) δ 38.1, 52.1, 68.2, 70.1, 72.0, 129.2, 130.3, 131.3, 134.1, 134.2, 137.1, 172.1; HRMS (ESI-TOF) Calcd. for C_14_H_16_NO_3_ [*M* + H]^+^ 246.1130. Found 246.1168.
Geometry. All e.s.d.'s (except the e.s.d. in the dihedral angle between two l.s. planes) are estimated using the full covariance matrix. The cell e.s.d.'s are taken into account individually in the estimation of e.s.d.'s in distances, angles and torsion angles; correlations between e.s.d.'s in cell parameters are only used when they are defined by crystal symmetry. An approximate (isotropic) treatment of cell e.s.d.'s is used for estimating e.s.d.'s involving l.s. planes.
Refinement. Refinement of *F*^2^ against ALL reflections. The weighted *R*-factor *wR* and goodness of fit *S* are based on *F*^2^, conventional *R*-factors *R* are based on *F*, with *F* set to zero for negative *F*^2^. The threshold expression of *F*^2^ > σ(*F*^2^) is used only for calculating *R*-factors(gt) *etc*. and is not relevant to the choice of reflections for refinement. *R*-factors based on *F*^2^ are statistically about twice as large as those based on *F*, and *R*- factors based on ALL data will be even larger.

### Fractional atomic coordinates and isotropic or equivalent isotropic displacement parameters (Å^2^)

**Table:**

	*x*	*y*	*z*	*U*_iso_*/*U*_eq_	
O1	0.2407 (3)	0.95636 (13)	0.17238 (19)	0.1020 (7)	
H1	0.1725	1.0026	0.1962	0.153*	
O2	0.52812 (19)	0.71064 (10)	0.18954 (10)	0.0546 (3)	
O3	−0.14247 (18)	0.65007 (11)	0.28956 (12)	0.0615 (4)	
H3	−0.2555	0.6653	0.2643	0.092*	
N1	0.2098 (2)	0.75095 (9)	0.13314 (9)	0.0364 (3)	
C6	0.1266 (3)	0.91174 (13)	0.09912 (14)	0.0478 (4)	
H1A	0.0930	0.9587	0.0460	0.057*	
C5	0.2537 (3)	0.82646 (13)	0.06255 (12)	0.0465 (4)	
H2A	0.2099	0.8063	−0.0030	0.056*	
H2B	0.4021	0.8428	0.0614	0.056*	
C3	0.3405 (2)	0.71615 (11)	0.20026 (12)	0.0364 (3)	
C2	0.2186 (2)	0.68831 (11)	0.28681 (11)	0.0336 (3)	
C4	0.3095 (2)	0.64180 (11)	0.36077 (11)	0.0381 (3)	
H5	0.4483	0.6237	0.3490	0.046*	
C8	0.2326 (3)	0.61406 (11)	0.45626 (11)	0.0389 (4)	
C13	0.3573 (3)	0.55253 (13)	0.51150 (13)	0.0498 (4)	
H7	0.4864	0.5321	0.4864	0.060*	
C12	0.2964 (4)	0.52072 (15)	0.60216 (14)	0.0635 (6)	
H8	0.3822	0.4779	0.6382	0.076*	
C11	0.1114 (4)	0.55125 (15)	0.63990 (13)	0.0631 (6)	
H9	0.0687	0.5294	0.7019	0.076*	
C7A	−0.0026 (2)	0.75856 (12)	0.16618 (11)	0.0378 (3)	
H10	−0.0881	0.7114	0.1283	0.045*	
C1	0.0020 (2)	0.72470 (11)	0.27237 (11)	0.0364 (3)	
H11	−0.0253	0.7814	0.3162	0.044*	
C10	−0.0113 (4)	0.61354 (15)	0.58734 (13)	0.0603 (5)	
H12	−0.1380	0.6353	0.6140	0.072*	
C9	0.0468 (3)	0.64509 (14)	0.49607 (13)	0.0498 (4)	
H13	−0.0401	0.6879	0.4607	0.060*	
C7	−0.0656 (3)	0.86200 (15)	0.13809 (16)	0.0584 (5)	
H14A	−0.1744	0.8603	0.0880	0.070*	
H14B	−0.1189	0.8977	0.1951	0.070*	

### Atomic displacement parameters (Å^2^)

**Table:**

	*U*^11^	*U*^22^	*U*^33^	*U*^12^	*U*^13^	*U*^23^
O1	0.0789 (13)	0.0675 (10)	0.159 (2)	0.0143 (9)	−0.0431 (13)	−0.0432 (12)
O2	0.0280 (6)	0.0762 (9)	0.0597 (7)	0.0084 (6)	0.0039 (5)	0.0184 (7)
O3	0.0281 (6)	0.0713 (9)	0.0852 (10)	−0.0097 (6)	−0.0096 (6)	0.0430 (8)
N1	0.0322 (6)	0.0392 (6)	0.0379 (6)	0.0041 (5)	−0.0002 (5)	0.0048 (5)
C6	0.0442 (9)	0.0423 (8)	0.0570 (10)	0.0035 (7)	−0.0013 (8)	0.0138 (8)
C5	0.0410 (10)	0.0574 (10)	0.0411 (8)	0.0042 (7)	0.0057 (7)	0.0141 (7)
C3	0.0278 (8)	0.0392 (8)	0.0421 (8)	0.0037 (6)	−0.0011 (6)	0.0029 (6)
C2	0.0292 (7)	0.0355 (7)	0.0361 (7)	−0.0007 (6)	−0.0034 (6)	0.0018 (6)
C4	0.0309 (7)	0.0418 (8)	0.0418 (8)	−0.0003 (6)	−0.0042 (6)	0.0033 (7)
C8	0.0451 (9)	0.0348 (7)	0.0367 (8)	−0.0039 (7)	−0.0068 (7)	−0.0012 (6)
C13	0.0540 (11)	0.0485 (10)	0.0469 (9)	0.0012 (8)	−0.0085 (8)	0.0047 (8)
C12	0.0888 (16)	0.0570 (11)	0.0448 (10)	−0.0003 (11)	−0.0128 (11)	0.0131 (9)
C11	0.0951 (16)	0.0564 (11)	0.0377 (9)	−0.0114 (11)	0.0023 (10)	0.0015 (8)
C7A	0.0255 (7)	0.0454 (8)	0.0425 (8)	−0.0020 (6)	−0.0051 (6)	0.0094 (6)
C1	0.0283 (7)	0.0387 (7)	0.0420 (8)	0.0030 (6)	0.0001 (6)	0.0077 (6)
C10	0.0782 (14)	0.0562 (10)	0.0464 (9)	0.0000 (11)	0.0177 (10)	−0.0107 (8)
C9	0.0599 (11)	0.0458 (9)	0.0437 (9)	0.0058 (8)	0.0036 (8)	−0.0026 (7)
C7	0.0440 (10)	0.0594 (11)	0.0717 (12)	0.0174 (8)	0.0097 (9)	0.0263 (10)

### Geometric parameters (Å, º)

**Table:**

O1—C6	1.397 (3)	C8—C9	1.393 (3)
O1—H1	0.8400	C8—C13	1.397 (2)
O2—C3	1.231 (2)	C13—C12	1.386 (3)
O3—C1	1.4074 (19)	C13—H7	0.9500
O3—H3	0.8400	C12—C11	1.376 (4)
N1—C3	1.346 (2)	C12—H8	0.9500
N1—C5	1.450 (2)	C11—C10	1.375 (3)
N1—C7A	1.458 (2)	C11—H9	0.9500
C6—C5	1.517 (2)	C7A—C7	1.524 (2)
C6—C7	1.521 (3)	C7A—C1	1.541 (2)
C6—H1A	1.0000	C7A—H10	1.0000
C5—H2A	0.9900	C1—H11	1.0000
C5—H2B	0.9900	C10—C9	1.387 (3)
C3—C2	1.486 (2)	C10—H12	0.9500
C2—C4	1.342 (2)	C9—H13	0.9500
C2—C1	1.507 (2)	C7—H14A	0.9900
C4—C8	1.463 (2)	C7—H14B	0.9900
C4—H5	0.9500		
			
C6—O1—H1	109.5	C8—C13—H7	119.4
C1—O3—H3	109.5	C11—C12—C13	119.9 (2)
C3—N1—C5	126.33 (14)	C11—C12—H8	120.1
C3—N1—C7A	113.99 (12)	C13—C12—H8	120.1
C5—N1—C7A	110.30 (12)	C10—C11—C12	119.62 (18)
O1—C6—C5	106.77 (16)	C10—C11—H9	120.2
O1—C6—C7	111.99 (19)	C12—C11—H9	120.2
C5—C6—C7	102.82 (14)	N1—C7A—C7	103.95 (13)
O1—C6—H1A	111.6	N1—C7A—C1	105.03 (12)
C5—C6—H1A	111.6	C7—C7A—C1	121.85 (16)
C7—C6—H1A	111.6	N1—C7A—H10	108.4
N1—C5—C6	102.46 (13)	C7—C7A—H10	108.4
N1—C5—H2A	111.3	C1—C7A—H10	108.4
C6—C5—H2A	111.3	O3—C1—C2	111.20 (12)
N1—C5—H2B	111.3	O3—C1—C7A	111.48 (13)
C6—C5—H2B	111.3	C2—C1—C7A	104.12 (12)
H2A—C5—H2B	109.2	O3—C1—H11	110.0
O2—C3—N1	124.34 (16)	C2—C1—H11	110.0
O2—C3—C2	127.58 (15)	C7A—C1—H11	110.0
N1—C3—C2	108.08 (12)	C11—C10—C9	121.1 (2)
C4—C2—C3	120.10 (14)	C11—C10—H12	119.5
C4—C2—C1	132.00 (14)	C9—C10—H12	119.5
C3—C2—C1	107.86 (12)	C10—C9—C8	120.10 (19)
C2—C4—C8	131.40 (15)	C10—C9—H13	120.0
C2—C4—H5	114.3	C8—C9—H13	120.0
C8—C4—H5	114.3	C6—C7—C7A	106.55 (14)
C9—C8—C13	118.05 (16)	C6—C7—H14A	110.4
C9—C8—C4	125.05 (15)	C7A—C7—H14A	110.4
C13—C8—C4	116.90 (16)	C6—C7—H14B	110.4
C12—C13—C8	121.3 (2)	C7A—C7—H14B	110.4
C12—C13—H7	119.4	H14A—C7—H14B	108.6
			
C3—N1—C5—C6	−109.15 (17)	C3—N1—C7A—C7	130.55 (16)
C7A—N1—C5—C6	34.96 (17)	C5—N1—C7A—C7	−18.33 (18)
O1—C6—C5—N1	81.68 (19)	C3—N1—C7A—C1	1.52 (18)
C7—C6—C5—N1	−36.33 (18)	C5—N1—C7A—C1	−147.35 (13)
C5—N1—C3—O2	−31.5 (3)	C4—C2—C1—O3	−52.6 (2)
C7A—N1—C3—O2	−174.55 (16)	C3—C2—C1—O3	129.73 (14)
C5—N1—C3—C2	147.63 (14)	C4—C2—C1—C7A	−172.77 (17)
C7A—N1—C3—C2	4.63 (18)	C3—C2—C1—C7A	9.56 (16)
O2—C3—C2—C4	−7.9 (3)	N1—C7A—C1—O3	−126.78 (14)
N1—C3—C2—C4	172.96 (14)	C7—C7A—C1—O3	115.79 (17)
O2—C3—C2—C1	170.10 (17)	N1—C7A—C1—C2	−6.80 (16)
N1—C3—C2—C1	−9.04 (17)	C7—C7A—C1—C2	−124.23 (16)
C3—C2—C4—C8	173.90 (15)	C12—C11—C10—C9	1.0 (3)
C1—C2—C4—C8	−3.5 (3)	C11—C10—C9—C8	−0.3 (3)
C2—C4—C8—C9	−10.3 (3)	C13—C8—C9—C10	−1.1 (3)
C2—C4—C8—C13	170.38 (17)	C4—C8—C9—C10	179.60 (17)
C9—C8—C13—C12	1.8 (3)	O1—C6—C7—C7A	−88.1 (2)
C4—C8—C13—C12	−178.83 (17)	C5—C6—C7—C7A	26.1 (2)
C8—C13—C12—C11	−1.1 (3)	N1—C7A—C7—C6	−5.9 (2)
C13—C12—C11—C10	−0.3 (3)	C1—C7A—C7—C6	112.09 (18)

### Hydrogen-bond geometry (Å, º)

**Table:**

*D*—H···*A*	*D*—H	H···*A*	*D*···*A*	*D*—H···*A*
O1—H1···O3^i^	0.84	2.04	2.776 (2)	147
O3—H3···O2^ii^	0.84	1.85	2.6810 (17)	168

Symmetry codes: (i) −*x*, *y*+1/2, −*z*+1/2; (ii) *x*−1, *y*, *z*.
